# Studies of *CTNNBL1 *and *FDFT1 *variants and measures of obesity: analyses of quantitative traits and case-control studies in 18,014 Danes

**DOI:** 10.1186/1471-2350-10-17

**Published:** 2009-02-26

**Authors:** Camilla Helene Andreasen, Mette Sloth Mogensen, Knut Borch-Johnsen, Annelli Sandbæk, Torsten Lauritzen, Katrine Almind, Lars Hansen, Torben Jørgensen, Oluf Pedersen, Torben Hansen

**Affiliations:** 1 Steno Diabetes Center, 2820 Gentofte, Denmark; 2 Novo Nordisk A/S, Medical and Science, Development Projects, 2880 Bagsværd, Denmark; 3 Faculty of Health Science, University of Aarhus, 8000 Aarhus, Denmark; 4 Department of General Practice, University of Aarhus, 8000 Aarhus, Denmark; 5 Bristol-Meyers Squibb & Co., Discovery Medicine and Clinical Pharmacology, CV Metabolic Diseases, 08543-4000, Princeton, NJ, USA; 6 Faculty of Health Sciences, University of Copenhagen, 2200 Copenhagen, Denmark; 7 Research Centre for Prevention and Health, Glostrup University Hospital, 2600 Glostrup, Denmark; 8 Faculty of Health Sciences, University of Southern Denmark, 5000 Odense, Denmark

## Abstract

**Background:**

A genome-wide scan in unrelated US Caucasians identified rs7001819 upstream of farnesyl-diphosphate farnesyltransferase 1 (*FDFT1*) and multiple variants within catenin (cadherin-associated protein), β-like 1 (*CTNNBL1*) to associate strongly with body mass index (BMI). The most significantly associating variants within *CTNNBL1 *including rs6013029 and rs6020846 were additionally confirmed to associate with morbid obesity in a French Caucasian case-control sample. The aim of this study was to investigate the impact of these three variants on obesity, through analyses of obesity-related quantitative traits, and case-control studies in large study samples of Danes.

**Methods:**

The *FDFT1 *rs7001819, *CTNNBL1 *rs6013029 and rs6020846 were genotyped, using TaqMan allelic discrimination, in a combined study sample comprising 18,014 participants ascertained from; the population-based Inter99 cohort (*n *= 6,514), the ADDITION Denmark screening study cohort (*n *= 8,662), and a population-based sample (*n *= 680) and a type 2 diabetic patients group (*n *= 2,158) from Steno Diabetes Center.

**Results:**

Both *CTNNBL1 *variants associated with body weight and height with per allele effect sizes of 1.0 [0.3–0.8] kg and 0.6 [0.2–0.9] cm, respectively, for the rs6020846 G-allele. No association was observed with BMI and waist circumference. In case-control studies neither of the *CTNNBL1 *variants showed association with overweight, obesity or morbid obesity (rs6013029: Odds Ratio (OR)_overweight _= 1.02 [0.90–1.16], OR_obesity _= 1.09 [0.95–1.25], OR_morbidobesity _= 1.26 [0.91–1.74]; rs6020846: OR_overweight _= 1.05 [0.93–1.18], OR_obesity_= 1.13 [1.00–1.28], OR_morbidobesity _= 1.17 [0.86–1.61]). However, in meta-analyses of the present and the previous study, both the rs6013029 T-allele and the rs6020846 G-allele increased the risk of developing morbid obesity (rs6013029: OR_combined _= 1.36 [1.12–1.64], *p *= 0.002; rs6020846: OR_combined _= 1.26 [1.06–1.51], *p *= 0.01), and obesity (rs6013029: OR_combined _= 1.17 [1.04–1.31], *p *= 0.007; rs6020846: OR_combined _= 1.17 [1.05–1.30], *p *= 0.004).

The *FDFT1 *rs7001819 C-allele showed no association with obesity-related quantitative measures or dichotomous measures of overweight, obesity and morbid obesity.

**Conclusion:**

*CTNNBL1 *variants associated with body weight and height, and confer the risk of developing obesity in meta-analyses combining the present and a previous study. *FDFT1 *rs7001819 showed no association with obesity, neither when analysing quantitative traits nor when performing case-control studies of obesity.

## Background

As a consequence of a changing environment and lifestyle, the rates of obesity, are escalating throughout the world. This poses a major problem towards human health through the association with increased risk of several chronic diseases, such as type 2 diabetes, hypertension and cardiovascular diseases, leading to premature mortality. Since obesity results from a complex interplay between several genetic and environmental risk factors, the identification of single nucleotide polymorphisms (SNPs) in susceptibility genes has been challenging using traditional approaches, such as linkage scans and biological candidate gene association studies, leaving the genetic background of common obesity largely unknown. Technical advancements have, however, made genome-wide association (GWA) studies feasible, which uses a hypothesis-free approach to scan the entire human genome for genetic variants associated with disease. These studies have led to a breakthrough in the identification of novel gene loci with modest effects on complex diseases.

Several GWA scans have been performed on obesity and obesity-related phenotypes, and the most frequently identified gene loci is *FTO *[1-4], which has been consistently replicated in numerous independent association studies [5-14]. Other GWA studies have reported variants in the proximity of *INSIG2 *[15,16] and within *PFKP *[2,16] as novel obesity gene loci, however, attempts to replicate the association for the *INSIG2 *variant have been inconsistent [17-24], which also appears true for variation in *PFKP *[17]. A recent initiative in the identification of obesity susceptibility genes is large-scale meta-analyses combining data from different GWA studies. This has lead to the identification of genetic variants downstream of *MC4R*, exerting effects on body mass index (BMI), and waist circumference, which was further supported by an independent GWA study [25].

A recent GWA scan in 1,000 US Caucasians with measures available on BMI and fat mass, identified two novel obesity gene loci in the proximity of *FDFT1 *(farnesyl-diphosphate farnesyltransferase 1) and *CTNNBL1 *(catenin (cadherin-associated protein), β-like 1) [16]. The strongest association with BMI was observed for rs7001819 (*p *= 2 × 10^-7^) mapping approximately 1 kb upstream of *FDFT1*, however, no association with fat mass was shown, and therefore no further analyses were performed for this variant. Eight SNPs within *CTNNBL1 *showed strong associations with both BMI and fat mass, out of which five (rs6013029, rs16986921, rs6020712, rs6020846, rs6020395) were replicated in an independent French Caucasian obesity case-control sample comprising 896 cases (BMI ≥ 40 kg/m^2^) and 2,916 controls (BMI < 25 kg/m^2^). In both study groups rs6013029 exerted the largest effect, conferring a per risk T-allele increase of 2.67 kg/m^2 ^in BMI and 5.96 kg in fat mass, and a 1.42-fold increased odds of morbid obesity comparing homozygous T-allele carriers with homozygous G-allele carriers.

The link between *FDFT1*, *CTNNBL1 *and obesity is fairly unknown. *FDFT1 *encodes the enzyme catalysing the first step of cholesterol synthesis [26], whereas *CTNNBL1 *encodes a protein homolog to β-catenin responsible for cell-to-cell adhesion and Wnt-signalling [27-29]. Expression profiles in the human protein atlas  show that *FDFT1 *predominantly is expressed in the colon, whereas *CTNNBL1 *is ubiquitously expressed. Moreover, studies have revealed that *CTNNBL1 *is highly expressed in skeletal muscle responsible for energy metabolism [27]. Linkage scans of obesity have previously highlighted the chromosome 20q11 region, which encompasses *CTNNBL1*, as a candidate region [30,31], thus, further establishing the candidature of this gene in the pathogenesis of obesity.

Replication of GWA findings in well-designed and statistically powered studies is essential to elucidate the importance of the proposed findings on obesity. Therefore, in the present study, we genotyped rs7001819 near *FDFT1 *responsible for the strongest BMI association and two tagSNPs (rs6020846 and rs6013029) that according to HapMap capture (*r*^2^cutoff = 0.8) the five replicated SNPs in *CTNNBL1 *identified in the initial GWA study. The aim was to elucidate whether the three variants associated with obesity in large study samples of Danes, by examining obesity-related quantitative traits, and by performing case-control studies of overweight, obesity and morbid obesity.

## Methods

### Subjects

The *FDFT1 *rs7001819, *CTNNBL1 *rs6013029 and rs6020846 variants were genotyped in 18,014 individuals ascertained from four different study groups; 1) the Inter99 cohort, which is a population-based, randomized, non-pharmacological intervention study of middle-aged individuals for the prevention of ischemic heart disease (*n *= 6,514), conducted at the Research Centre for Prevention and Health in Glostrup, Copenhagen (ClinicalTrials.gov ID-no: NCT00289237) [32,33]; 2) the ADDITION Denmark screening study cohort (Anglo-Danish-Dutch Study of Intensive Treatment in People with Screen-Detected Diabetes in Primary Care) (ClinicalTrials.gov ID-no: NCT00237548) [34], which is a population-based, high-risk screening and intervention study for type 2 diabetes in general practice (*n*= 8,662); 3) a population-based group of unrelated middle-aged individuals (*n *= 680) examined at Steno Diabetes Center; and 4) unrelated type 2 diabetic patients (*n *= 2,158) sampled through the out-patient clinic at Steno Diabetes Center. In the combined study sample 1,914 had screen-detected and 2,302 had known type 2 diabetes, 5,512 were normal weight, 7,458 were overweight and 5,044 were obese, with 409 being morbidly obese. All participants in study group 1 and 3 underwent a standard 75 g oral glucose tolerance test. Type 2 diabetes and glucose tolerance were defined according to the World Health Organization [35]. Overweight, obesity and morbid obesity were defined as 25 kg/m^2 ^≤ BMI < 30 kg/m^2^, BMI ≥ 30 kg/m^2 ^and BMI ≥ 40 kg/m^2^, respectively. More information about the study groups is available in Additional file 1. All study participants were Danes by self-report, and informed written consent was obtained from all subjects before participation. The studies were approved by the regional Ethical Committees (ethics committee, Copenhagen County for study group 1, 3 and 4 and ethics committee, Aarhus County for study group 2) and were in accordance with the principles of the *Helsinki Declaration*.

### Biochemical and anthropometric measures

In all four study groups body weight and height were measured in light indoor clothes and without shoes. BMI was defined as weight in kilograms divided by height in meters squared (kg/m^2^). Waist circumference (cm) was measured with subjects in standing position midway between the iliac crest and the lower costal margin. In study group 1, 3 and 4 blood samples were drawn after a 12-hour overnight fast and total cholesterol and high density lipoprotein (HDL) -cholesterol were determined using enzymatic colorimetric methods (GPO-PAP and CHOD-PAP; Roche Molecular Biochemicals, Mannheim, Germany). In study group 2 fasting blood glucose was measured on capillary whole blood using a HemoCue B-glucose analyser (HemoCue AB, Ängelholm, Sweden) and the Hitachi 971 system (Roche Diagnostics GmbH, Mannheim, Germany) was used to measure total serum cholesterol and serum HDL-cholesterol.

### Genotyping

*FDFT1 *rs7001819, *CTNNBL1 *rs6013029 and rs6020846 were genotyped in 18,014 Danes using TaqMan allelic discrimination (KBioscience, Herts, UK). Genotyping success rates were 93.1%, 97.3% and 97.1%, respectively, and the minor allele frequencies (MAF) were 36.1%, 4.6% and 5.4%, respectively. The discordance rates between 1,185, 1,170 and 1,170 random duplicate samples were 0.42%, 0.17% and 0.43%, respectively. Genotype distribution obeyed Hardy-Weinberg equilibrium for all three variants *p *= 0.3, *p *= 0.06 and *p *= 0.5, respectively.

### Statistical analysis

Homogeneity in the combined study sample, including the population-based Inter99 cohort, the ADDITION Denmark screening study cohort, the population-based and type 2 diabetic patient groups from Steno Diabetes Center, was evaluated for each of the three variants. Homogeneity tests were performed by means of the Mantel-Haenszel method (fixed effect model) and revealed no statistically significant heterogeneity between study groups (rs7001819: *p *= 0.7, rs6013029: *p *= 1.0, rs6020846: *p *= 0.8).

To test quantitative obesity-related traits for differences between genotype groups a general linear model was used, applying an additive and dominant model for each variant and including adjustments for sex, age and BMI when appropriate. Case-control studies of overweight, obesity and morbid obesity were performed in the combined study sample and separately in the population-based Inter99 study sample, by applying logistic regression to examine differences in genotype distributions between affected and unaffected subjects. An additive and dominant model was applied and adjustments for sex and age were introduced. Meta-analyses of the present and the previous study were performed using allele frequencies, applying general linear models with the Mantel-Haenszel method. All analyses were performed in RGui version 2.6.2 [36], and *p*-values < 0.05 were considered significant. Linkage Disequilibrium (LD) between markers was evaluated using Haploview 4.1 . Statistical power for the quantitative traits was estimated using simulations (*n *= 5,000), where variance across genotypes were drawn from phenotypes simulated to follow normal distribution using empirical variances. The variance for adjustment factors, estimated using residuals of linear models, was also included in the model, assuming independency of genotypes. Linear models were used both for simulating and testing data, assuming additive models and using a significance threshold of 0.05. The estimated power for variants with a MAF of 5% was > 90% for per allele effect sizes of 0.4 kg/m^2 ^for BMI and 1.5 cm for waist circumference.

Statistical power in case-control settings was determined using the CaTS power calculator version 0.0.2. Using the population-based Inter99 cohort as reference the prevalence of overweight, obesity and morbid obesity in the Danish population was estimated to 39%, 17% and 1.3%, respectively. This gives us a statistical power of 100% and 94% to detect associations with overweight and obesity, respectively, for a variant with a MAF of 5% with a relative risk of 1.2 and a statistical power of 53% to detect association with morbid obesity, for a variant with a MAF of 5% with a relative risk of 1.4.

## Results

We aimed at replicating the observed association between the *CTNNBL1 *rs6013029 T-allele, *CTNNBL1 *rs6020846 G-allele, and *FDFT1 *rs7001819 C-allele and measures of obesity. The variants were investigated for influence on quantitative obesity-related traits in the combined study sample, excluding treated type 2 diabetic patients. Moreover, case-controls settings were used to investigate potential associations between the variants and overweight, obesity and morbid obesity in the combined study sample including type 2 diabetic patients.

**Figure 1:**
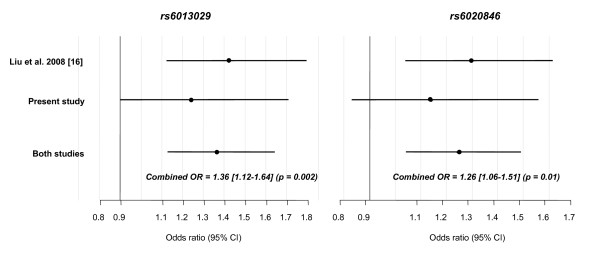
**Meta-analyses of morbid obesity for *CTNNBL1 *rs6013029 and rs6020846**. Estimated odds ratios (OR) [95% CI] for morbid obesity for minor allele carriers of *CTNNBL1 *rs6013029 and rs6020846, in combined analyses of the present, and a previous study [16]. No heterogeneity between the two studies was observed (*p *= 0.5). The study by Liu et al. [16] included 2,669 lean controls and 877 morbidly obese cases for both variants and the present study included 5,190 controls and 399 cases for rs6013029 and 5,188 controls and 394 cases for rs6020846.

The LD between the two *CTNNBL1 *variants was estimated to *r*^2 ^= 0.83 in the combined study sample. Neither variant showed association with BMI nor waist circumference, however, the rs6013029 T-allele and rs6020846 G-allele associated with body weight (rs6013029: *p *= 0.03; rs6020846: *p *= 0.007) and height (rs6013029: *p *= 0.007; rs6020846: *p *= 5 × 10^-4^), with rs6020846 exerting the largest effect, with a per G-allele increase in body weight of 1.0 [0.3–1.8] kg and in height of 0.6 [0.2–0.9] cm, Table 1. In case-control studies no statistically significant associations were observed when comparing the genotype distribution between normal weight individuals and overweight, obese or morbid obese individuals (rs6013029: Odds Ratio (OR)_overweight _= 1.02 [0.90–1.16], OR_obesity _= 1.09 [0.95–1.25], OR_morbidobesity _= 1.26 [0.91–1.74]; rs6020846: OR_overweight _= 1.05 [0.93–1.18], OR_obesity _= 1.13 [1.00–1.28], OR_morbidobesity _= 1.17 [0.86–1.61]), Table 2. However, for both variants, the risk allele frequency increased with increasing degree of obesity, also reflected in the relatively large effect sizes estimated by the ORs, which are per allele increases in risk. We therefore performed meta-analyses, based on available allele frequencies, combining our data with data from the French Caucasian obesity case-control sample used for replication in the initial study by Liu et al. [16]. Both *CTNNBL1 *variants associated with increased susceptibility to morbid obesity (rs6013029: OR_combined _= 1.36 [1.12–1.64], *p *= 0.002; rs6020846: OR_combined _= 1.26 [1.06–1.51], *p *= 0.01), Figure 1. Test of between-study homogeneity showed no heterogeneity for either rs6013029 (*p *= 0.5) or rs6020846 (*p *= 0.5). Moreover, a meta-analysis combining our data on obese individuals with the French Caucasian morbid obesity data, also showed significant associations for both variants (rs6013029: OR_combined _= 1.17 [1.04–1.31], *p *= 0.007; rs6020846: OR_combined _= 1.17 [1.05–1.30], *p *= 0.004), with no between-study heterogeneity (*p *= 0.1 and 0.2 respectively).

**Table 1:** Quantitative obesity-related measures in the combined study sample

		***n *** **(men/woman) **	**Age ** **(years)**	**BMI ** **(kg/m^2^)**	**Body weight ** **(kg)**	**Waist circumference ** **(cm)**	**Height ** **(cm)**
*CTNNBL1 *rs6013029	GG	13,579 (7,113/6,466)	54 ± 10	27.5 ± 4.8	81.0 ± 16.2	92.5 ± 14.3	171.2 ± 9.2
	GT	1,318 (702/616)	55 ± 10	27.7 ± 4.9	82.0 ± 16.5	93.1 ± 14.5	171.8 ± 9.1
	TT	22 (9/13)	56 ± 9	27.6 ± 4.5	80.2 ± 12.5	91.9 ± 11.9	170.8 ± 7.3
	Per allele effect size (95% CI)			0.16 (-0.10–0.42)	0.89 (0.08–1.69)	0.43 (-0.26–1.12)	0.48 (0.13–0.82)
	*P*_add_			0.2	0.03	0.2	0.007
	*P*_dom_			0.2	0.03	0.2	0.009
*CTNNBL1 *rs6020846	AA	13,317 (6,972/6,345)	54 ± 10	27.5 ± 4.8	80.9 ± 16.2	92.5 ± 14.3	171.2 ± 9.2
	AG	1,527 (821/706)	55 ± 10	27.7 ± 4.9	82.1 ± 16.5	93.2 ± 14.3	171.9 ± 9.1
	GG	42 (23/19)	57 ± 9	27.8 ± 4.5	83.7 ± 15.4	94.5 ± 14.0	173.2 ± 8.4
	Per allele effect size (95% CI)			0.17 (-0.07–0.41)	1.02 (0.28–1.76)	0.54 (-0.09–1.17)	0.56 (0.25–0.88)
	*P*_add_			0.2	0.007	0.1	5 × 10^-4^
	*P*_dom_			0.2	0.008	0.1	0.001
*FDFT1* rs7001819	TT	5,929 (3,062/2,867)	55 ± 10	27.5 ± 4.9	80.8 ± 16.3	92.4 ± 14.4	171.1 ± 9.2
	TC	6,823 (3,597/3,226)	54 ± 10	28.0 ± 4.9	81.3 ± 16.4	92.4 ± 14.4	171.5 ± 9.3
	CC	1,886 (1,021/865)	55 ± 10	27.5 ± 4.8	81.0 ± 15.8	92.6 ± 13.9	171.3 ± 9.1
	Per allele effect size (95% CI)			0.01 (-0.10–0.13)	0.06 (-0.29–0.41)	0.03 (-0.28–0.33)	0.06 (-0.09–0.21)
	*P*_add_			0.8	0.8	0.9	0.4
	*P*_dom_			0.6	0.3	0.5	0.07

Data are means ± standard deviation. *p*-values were calculated assuming a additive (*p*_add_) and a dominant (*p*_dom_) model for all variants. Known type 2 diabetic patients were excluded from the analyses. Per allele effect sizes was calculated using linear models assuming an additive model. Adjustments were made for the effect of age and sex.

**Table 2:** Case-control studies of overweight, obesity and morbid obesity in the combined study sample

***CTNNBL1 *rs6013029**	***n *** **(men/women)**	**Genotype distribution ** ***n *GG/GT/TT (%)**	**MAF ** **(95% CI)**	***p*_add_**	***p*_dom_**	**OR_add _** **(95% CI)**
**Controls**	5,190 (2,271/3,019)	4,740/444/6 (91.4/8.5/0.1)	4.4 (4.0–4.8)			
**Overweight cases**	7,229 (4,496/2,733)	6,589/629//11 (91.1/8.7/0.2)	4.5 (4.2–4.9)	0.7	0.8	1.02 (0.90–1.16)
**Obese cases**	4,928 (2,543/2,385)	4,464/456/8 (90.5/9.3/0.2)	4.8 (4.4–5.2)	0.2	0.2	1.09 (0.95–1.25)
**Morbid obese cases**	399 (135/264)	356/43/0 (89.1/10.9/0.0)	5.4 (3.9–7.2)	0.2	0.1	1.26 (0.91–1.74)
***CTNNBL1 *rs6020846**	***n*** **(men/women)**	**Genotype distribution ** ***n *AA/GA/GG (%)**	**MAF** **(95% CI)**	***p*_add_**	***p*_dom_**	**OR_add_** **(95% CI)**
**Controls**	5,188 (2,169/3,019)	4,673/504/11 (90.1/9.7/0.2)	5.1 (4.7–5.5)			
**Overweight cases**	7,204 (4,492/2,712)	6,448/738/18 (89.4/10.3/0.2)	5.4 (5.0–5.8)	0.5	0.5	1.05 (0.93–1.18)
**Obese cases**	4,905 (2,533/2,372)	4,358/531/16 (88.8/10.9/0.3)	5.7 (5.3–6.2)	0.06	0.06	1.13 (1.00–1.28)
**Morbid obese cases**	394 (134/260)	348/46/0 (88.2/11.8/0.0)	5.8 (4.3–7.7)	0.3	0.2	1.17 (0.86–1.61)
***FDFT1 *rs7001819**	***n*** **(men/women)**	**Genotype distribution ** ***n *TT/TC/CC (%)**	**MAF** **(95% CI)**	***p*_add_**	***p*_dom_**	**OR_add_** **(95% CI)**
**Controls**	5,089 (2,123/2,966)	2,084/2,367/638 (40.9/46.5/12.6)	35.8 (34.9–36.7)			
**Overweight cases**	7,078 (4,391/2,687)	2,843/3,281/954 (40.2/46.3/13.5)	36.7 (35.9–37.5)	0.5	0.6	1.02 (0.97–1.08)
**Obese cases**	4,810 (2,491/2,319)	1,963/2,258/589 (40.6/47.1/12.3)	35.7 (34.8–36.7)	0.6	0.9	0.99 (0.93–1.05)
**Morbid obese cases**	387 (131/256)	158/184/45 (41.0/47.5/11.5)	35.4 (32.0–38.9)	0.9	0.9	0.99 (0.85–1.16)

Data are number of subjects, divided into genotype groups (% in each group), frequencies of the minor allele (MAF) in percentages (95% CI) and odds ratio (OR) for an additive (add) model (95% CI). Since the *CTNNBL1 *variants are rather rare, the *p*-values are given for both an additive and dominant (dom) model. Differences in genotype distribution were evaluated using logistic regression. *p*-values were adjusted for age and sex. Controls were defined as BMI < 25 kg/m^2^, overweight cases as 25 kg/m^2 ^≤ BMI < 30 kg/m^2^, obese cases as BMI ≥ 30 kg/m^2 ^and morbid obese cases as BMI ≥ 40 kg/m^2^.

The *FDFT1 *rs7001819 C-allele showed no association with BMI, body weight, waist circumference or height, Table 1. Due to the involvement of *FDFT1 *in cholesterol biosynthesis we extended the analyses to include measures of total cholesterol and HDL-cholesterol, but no differences between genotype groups were observed (data not shown). Moreover, no association was observed with overweight, obesity or morbid obesity (OR_overweight _= 1.02 [0.97–1.08], OR_obesity _= 0.99 [0.93–1.05], OR_morbidobesity _= 0.99 [0.85–1.16]), Table 2.

To investigate the effect on a population-based level, analyses of obesity-related traits were moreover performed in the population-based Inter99 cohort. The association with body weight and height remained significant for *CTNNBL1 *rs6020846, while no associations were demonstrated for *FDFT1 *rs7001819, Additional file 2.

Likewise case-control studies in the population-based Inter99 cohort revealed no statistical association for any of the variants (*p*-values ranged from 0.2–1.0), but as in the combined study sample the rs6013029 T-allele and rs6020846 G-allele frequencies increased with the degree of obesity, Additional file 3. All three variants were analysed for influence on type 2 diabetes susceptibility in the combined study sample, however, no associations were observed, either with or without adjustments for BMI, Additional file 4.

## Discussion

We find that the *CTNNBL1 *rs6013029 T-allele and the rs6020846 G-allele confer an increased risk of developing obesity, especially morbid obesity. Moreover, significant associations with increased body weight and height were observed together with non-significant increases in the measures of BMI and waist circumference for carriers of the *CTNNBL1 *risk alleles. The *FDFT1 *rs7001819 C-allele, on the other hand, did not associate with measures of obesity, neither in quantitative analyses nor in case-control studies.

This is consistent with the results from Liu and co-workers [16], who in a GWA scan identified five SNPs in *CTNNBL1 *as novel obesity variants, associating with both BMI and fat mass as quantitative measures. These findings were confirmed in independent case-control studies of morbid obesity with a per allele OR ranking from 1.32–1.43. The degree of LD between rs6013029 and rs6020846 was higher in our combined study sample than estimated by HapMap (*r*^2 ^= 0.83 vs. 0.73), thus, the results for these two variants are highly concordant; with the rs6020846 G-allele exerting the largest effect on quantitative measures, and the rs6013029 T-allele on the risk of developing morbid obesity.

Both *CTNNBL1 *variants associated with body weight and height, with a per allele increase of 1.0 [0.3–1.8] kg and 0.6 [0.2–0.9] cm, respectively, for the rs6020846 G-allele. Likewise, we see increasing measures of BMI and waist circumference as a consequence of increasing number of risk alleles, however, not significant. The effect on BMI and waist circumference was 0.17 [-0.1–0.4] kg/m^2 ^and 0.5 [-0.1–1.2] cm, respectively, for the rs6020846 G-allele, which is smaller than the effects observed by Liu et al. [16]. Thus, the lack of statistical significant replication might be due to lack of power to detect these effect sizes.

In case-control settings rs6013029 we observed increasing MAF with increasing degree of obesity, with a per allele OR of 1.26 [0.91–1.74] when analysing morbid obesity, which is in the range of the OR observed by Liu et al. [16]. However, the increased risk did not reach statistical significance, probably due to the relatively low number of morbidly obese individuals in our study sample. Since our combined study sample is not designed to analyse morbid obesity, we are underpowered (< 90%) to detect an association with a relative risk below 1.65.

That variants in *CTNNBL1 *could confer risk to the development of morbid obesity is supported by the meta-analyses performed including the present study and the French Caucasian obesity case-control sample used for replication in the initial study [16]. Combining the two studies a significant overall association with a combined per allele OR of 1.36 [1.12–1.64] for the rs6020846 G-allele and 1.26 [1.06–1.51] for the rs6013029 T-allele was observed. Moreover, our analysis of obesity for rs6013029 showed borderline significance (*p *= 0.06) with a 1.13-fold per allele increase in the risk of developing obesity. When combining our data on obese individuals with the French Caucasian morbid obesity data used for replication by Liu et al. [16], in a meta-analysis, a significant association is also observed with a combined OR of 1.17 [1.04–1.31], suggesting that *CTNNBL1 *is likely be a true obesity gene conferring risk of developing both obesity and morbid obesity.

Regarding *FDFT1 *we failed to demonstrate an association between the rs7001819 C-allele and measures of obesity, both when investigating quantitative phenotypes including BMI and waist circumference and dichotomous BMI cut-offs of overweight, obesity and morbid obesity, consistent with the fact that no association with fat mass was observed in the initial study [16].

Investigating available GWA data on quantitative traits from the Broad Institute Diabetes Genetic Initiative (DGI; ), no association with body weight or height was observed for the two *CTNNBL1 *variants, either when looking at BMI and waist circumference [37]. Data for obesity case-control studies are unfortunately not available, and could therefore not be included in the present meta-analysis. *FDFT1 *rs7001819 was not included in the DGI data, neither were markers in high LD with the variant. The gene was represented by other markers, however, none of these showed any association with obesity-related quantitative traits [37], suggesting that variants at the *FDFT1 *locus do not associate with obesity.

From our studies we can exclude *FDFT1 *rs7001819 as a susceptibility variant conferring risk to obesity in the Danish population. Contrary, meta-analyses indicates that both the *CTNNBL1 *rs6013029 T-allele and the rs6020846 G-allele confer the risk of being obese and especially morbidly obese, however, large well-powered studies designed to analyse especially morbid obesity are needed to validate this observation.

## Conclusion

Both *CTNNBL1 *variants associated with body weight and height, whereas BMI and waist circumference was insignificantly elevated with increasing number of obesity risk alleles. Likewise, the MAF and OR increased with increasing degree of obesity in case-control studies of overweight, obesity and morbid obesity, and meta-analyses showed that both the rs6013029 T-allele and the rs6020846 G-allele significantly increases the risk of developing obesity, especially morbid obesity. The *FDFT1 *rs7001819 C-allele showed no association with neither quantitative nor dichotomous measures of obesity.

## Abbreviations

BMI: body mass index; CI: confidence interval; *CTNNBL1*: catenin (cadherin-associated protein), β-like 1; *FDFT1*: farnesyl-diphosphate farnesyltransferase 1; GWA: genome-wide association; LD: linkage disequilibrim; MAF: minor allele frequency; OR: odds ratio; SNP: single nucleotide polymorphism.

## Competing interests

CH Andreasen, MS Mogensen, K Borch-Johnsen, O Pedersen and T Hansen hold stock in Novo Nordisk and K Borch-Johnsen, O Pedersen and T Hansen have received lecture fees from pharmaceutical companies. All other authors declare that there is no competing interest associated with this manuscript.

## Authors' contributions

The concept and idea regarding the epidemiological studies underlying the study populations analysed in the genetic study was conceived by TJ, KBJ, AS, TL, OP and TH. The collection of study subjects was planned and performed by TJ, KBJ, AS, TL, OP and TH.

The original hypothesis regarding the genetic study was conceived by CHA and MSM and approved by OP and TH. Detail planning of analyses and study design was performed by CHA and MSM and approved by OP and TH. CHA, MSM, KA, LH, OP and TH contributed to the establishment of study population databases specific for this study. Statistical analyses in association studies were performed by CHA and MSM. The first manuscript was written by CHA and MSM with equal contribution and the final draft was finalised by CHA, OP and TH. All authors revised the manuscript and contributed to the discussion.

## Pre-publication history

The pre-publication history for this paper can be accessed here:



## Supplementary Material

Additional file 1 **Supplementary Table 1.** Subjects included in the analyses stratified according to study group. Click here for file

Additional file 2 **Supplementary Table 2**. Quantitative obesity-related measures in the population-based Inter99 cohort. Click here for file

Additional file 3 **Supplementary Table 3.** Case-control studies of overweight and obesity in the population-based Inter99 cohort. Click here for file

Additional file 4 **Supplementary Table 4.** DOC Case-control studies of type 2 diabetes in the combined study material. Click here for file
