# Supplementary material for: Studies of CTNNBL1 and FDFT1 variants and measures of obesity: analyses of quantitative traits and case-control studies in 18,014 Danes
Source: BMC Med Genet. 2009 Feb 26;10:17. doi: 10.1186/1471-2350-10-17 (PMC2669074; doi:10.1186/1471-2350-10-17)
Supplement: Additional file 1 — Supplementary Table 1. Subjects included in the analyses stratified according to study group. [file 1471-2350-10-17-S1.doc]

**Supplementary Table 1**

Subjects included in the analyses stratified according to study group

|  | Population-based Inter99 study sample | ADDITION Denmark screening study cohort | SDC population-based study group | SDC type 2 diabetes group |
| --- | --- | --- | --- | --- |
| Study group | 1 | 2 | 3 | 4 |
| n  (men/women) | 6,514  (3,169/3,345) | 8,662  (4,728/3,934) | 680  (339/341) | 2,158  (1,317/841) |
| Age (years) | 46.2 ± 7.9 | 60.0 ± 6.8 | 57.6 ± 9.4 | 62.1 ± 11.5 |
| Weight (kg) | 78.2 ± 16.2 | 83.5 ± 16.2 | 76.3 ±14.0 | 88.6 ± 18.8 |
| Height (cm) | 172.1 ± 9.2 | 170.7 ± 9.2 | 170.1 ± 9.0 | 171.5 ± 9.9 |
| BMI kg/m^2^ | 26.3 ± 4.6 | 28.6 ± 4.9 | 26.3 ± 4.2 | 30.1 ± 5.6 |
| Waist circumference (cm) | 86.7 ± 13.4 | 97.1 ± 13.5 | 88.3 ±12.3 | 103.7 ± 14.9 |

Data are means ± standard deviation.
