# Supplementary material for: Studies of CTNNBL1 and FDFT1 variants and measures of obesity: analyses of quantitative traits and case-control studies in 18,014 Danes
Source: BMC Med Genet. 2009 Feb 26;10:17. doi: 10.1186/1471-2350-10-17 (PMC2669074; doi:10.1186/1471-2350-10-17)
Supplement: Additional file 2 — Supplementary Table 2. Quantitative obesity-related measures in the population-based Inter99 cohort. [file 1471-2350-10-17-S2.doc]

**Supplementary Table 2**

Quantitative obesity-related measures in the population-based Inter99 cohort

|  |  | ***n***  **(men/woman)** | **Age**  **(years)** | **BMI**  **(kg/m^2^)** | **Body weight**  **(kg)** | **Waist circumference (cm)** | **Height**  **(cm)** |
| --- | --- | --- | --- | --- | --- | --- | --- |
| ***CTNNBL1* rs6013029** | **GG** | 5,275  (2,628/2,647) | 46 ± 8 | 26.2 ± 4.5 | 78.0 ± 16.0 | 86.4 ± 13.2 | 172.2 ± 9.1 |
|  | **GT** | 532  (259/273) | 47 ± 8 | 26.3 ± 4.7 | 78.7 ± 16.1 | 87.0 ± 13.2 | 172.8 ± 9.6 |
|  | **TT** | 8  (2/6) | 50 ± 9 | 26.9 ± 5.1 | 78.0 ± 13.9 | 83.9 ± 6.2 | 170.6 ± 6.9 |
|  | **Per allele effect size (95% CI)** |  |  | 0.08  (-0.31-0.47) | 0.90  (-0.32-2.13 | 0.52  (-0.46-1.51) | 0.82  (0.26-1.38) |
|  | ***p*_add_** |  |  | 0.7 | 0.2 | 0.3 | 0.004 |
|  | ***p*_dom_** |  |  | 0.7 | 0.2 | 0.3 | 0.005 |
| ***CTNNBL1* rs6020846** | **AA** | 5,195  (2,587/2,608) | 46 ± 8 | 26.2 ± 4.5 | 77.9 ± 15.9 | 86.4 ± 13.1 | 172.2 ± 9.1 |
|  | **AG** | 601  (298/303) | 47 ± 8 | 26.4 ± 4.7 | 79.3 ± 16.5 | 87.4 ± 13.3 | 172.9 ± 9.5 |
|  | **GG** | 14  (7/7) | 49 ± 7 | 26.5 ± 4.5 | 79.6 ± 15.7 | 86.5 ± 11.2 | 173.0 ± 8.7 |
|  | **Per allele effect size (95% CI)** |  |  | 0.21  (-0.15-0.57) | 1.31  (0.17-2.45) | 0.83  (-0.09-1.75) | 0.80  (0.28-1.32) |
|  | ***p*_add_** |  |  | 0.3 | 0.02 | 0.08 | 0.003 |
|  | ***p*_dom_** |  |  | 0.6 | 0.02 | 0.06 | 0.003 |
| ***FDFT1***  **rs7001819** | **TT** | 2,348  (1,126/1,222) | 46 ± 8 | 26.3 ± 4.7 | 77.8 ± 16.1 | 86.3 ± 13.3 | 171.9 ± 9.2 |
|  | **TC** | 2,678  (1,379/1,299) | 46 ± 8 | 26.2 ± 4.5 | 78.5 ± 16.0 | 86.7 ± 13.3 | 172.8 ± 9.3 |
|  | **CC** | 729  (361/368) | 46 ± 8 | 26.1 ± 4.4 | 77.9 ± 15.5 | 86.3 ± 12.7 | 172.1 ± 9.0 |
|  | **Per allele effect size (95% CI)** |  |  | -0.08  (-0.25-0.10) | -0.11  (-0.65-0.43) | -0.14  (-0.58-0.29) | 0.12  (-0.13-0.37) |
|  | ***p*_add_** |  |  | 0.4 | 0.7 | 0.5 | 0.4 |
|  | ***p*_dom_** |  |  | 0.6 | 0.3 | 0.5 | 0.07 |

Data are means ± standard deviation. *p*-values were calculated assuming a additive (*p*_add_) and a dominant (*p*_dom_) model for all variants. Known type 2 diabetic patients were excluded from the analyses. Per allele effect sizes was calculated using linear models assuming and additive model. Adjustments were made for the effect of age and sex.
