# Supplementary material for: Studies of CTNNBL1 and FDFT1 variants and measures of obesity: analyses of quantitative traits and case-control studies in 18,014 Danes
Source: BMC Med Genet. 2009 Feb 26;10:17. doi: 10.1186/1471-2350-10-17 (PMC2669074; doi:10.1186/1471-2350-10-17)
Supplement: Additional file 3 — Supplementary Table 3. Case-control studies of overweight and obesity in the population-based Inter99 cohort. [file 1471-2350-10-17-S3.doc]

**Supplementary Table 3**

**Case-control studies of overweight and obesity in the population-based Inter99 cohort**

| ***CTNNBL1* rs6013029** | ***n***  **(men/women)** | **Genotype distribution *n* GG/GT/TT (%)** | **MAF**  **(95% CI)** | ***p*_add_** | ***p*_dom_** | **OR_add_**  **(95% CI)** |
| --- | --- | --- | --- | --- | --- | --- |
| **Controls** | 2,712 (1.0492/1.670) | 2,463/245/4  (90.9/9.0/0.1) | 4.7  (4.1-5.3) |  |  |  |
| **Overweight cases** | 2,418  (1,461/957) | 2,194/222/2  (90.7/9.2/0.1) | 4.7  (4.1-5.3) | 1.0 | 1.0 | 1.0  (0.83-1.22) |
| **Obese cases** | 1,067 (525/542) | 969/96/2  (90.8/9.0/0.2) | 4.7  (3.8-5.7) | 0.9 | 0.8 | 0.97  (0.76-1.25) |
| ***CTNNBL1* rs6020846** | ***n***  **(men/women)** | **Genotype distribution *n* AA/GA/GG (%)** | **MAF**  **(95% CI)** | ***p*_add_** | ***p*_dom_** | **OR_add_**  **(95% CI)** |
| **Controls** | 2,717 (1,043/1,674) | 2,444/267/6  (90.0/9.8/0.2) | 5.1  (4.6-5.8) |  |  |  |
| **Overweight cases** | 2,410  (1,465/945) | 2,153/252/5  (89.3/10.5/0.2) | 5.4  (4.8-6.1) | 0.7 | 0.6 | 1.04  (0.87-1.25) |
| **Obese cases** | 1,061  (522/539) | 940/118/3  (88.8/10.9/0.3) | 5.8  (4.9-6.9) | 0.3 | 0.3 | 1.13  (0.89-1.42) |
| ***FDFT1* rs7001819** | ***n***  **(men/women)** | **Genotype distribution *n* TT/TC/CC (%)** | **MAF**  **(95% CI)** | ***p*_add_** | ***p*_dom_** | **OR_add_**  **(95% CI)** |
| **Controls** | 2,687 (1,031/1,656) | 1,121/1,237/329  (41.7/46.1/12.2) | 35.3  (34.0-36.6) |  |  |  |
| **Overweight cases** | 2,385  (1,442/942) | 954/1,110/321  (40.0/46.5/13.5) | 36.7  (35.4-38.1) | 0.2 | 0.4 | 1.05  (0.97-1.15) |
| **Obese cases** | 1,059  (527/532) | 442/497/120  (41.7/46.9/11.3) | 34.8  (32.8-36.9) | 0.5 | 0.8 | 0.96  (0.86-1.14) |

Data are number of subjects, divided into genotype groups (% in each group), frequencies of the minor allele (MAF) in percentages (95% CI) and odds ratio (OR) for an additive model (95% CI). Since the *CTNNBL1* variants are rather rare, the *p*-values are given for both an additive and dominant (dom) model. Differences in genotype distribution were evaluated using logistic regression. *p*-values were adjusted for age and sex. Controls were defined as BMI < 25 kg/m^2^, overweight cases as 25 kg/m^2^ ≤ BMI < 30 kg/m^2^, obese cases as BMI ≥ 30 kg/m^2^.
