# Supplementary material for: Studies of CTNNBL1 and FDFT1 variants and measures of obesity: analyses of quantitative traits and case-control studies in 18,014 Danes
Source: BMC Med Genet. 2009 Feb 26;10:17. doi: 10.1186/1471-2350-10-17 (PMC2669074; doi:10.1186/1471-2350-10-17)
Supplement: Additional file 4 — Supplementary Table 4. DOC Case-control studies of type 2 diabetes in the combined study material. [file 1471-2350-10-17-S4.doc]

**Supplementary Table 4**

Case-control studies of type 2 diabetes in the combined study material

| ***CTNNBL1***  **rs6013029** | ***n***  **(men/women)** | **Genotype distribution**  ***n* GG/GT/TT (%)** | **MAF**  **(95% CI)** | BMI adjustment | | No BMI adjustment | | **OR_add_**  **(95% CI)** |
| --- | --- | --- | --- | --- | --- | --- | --- | --- |
|  |  |  |  | ***p*_add_** | ***p*_dom_** | ***p*_add_** | ***p*_dom_** |  |
| **Controls** | 4,908  (2,283/2,626) | 4,464/439/6  (90.5/9.3/0.2) | 4.6  (4.2-5.0) |  |  |  |  |  |
| **T2D patients** | 3,582  (2,127/1,455) | 3,242/333/7  (91.0/8.9/0.1) | 4.8  (4.4-5.4) | 0.6 | 0.6 | 0.3 | 0.3 | 1.09  (0.91-1.31) |
| ***CTNNBL1***  **rs6020846** | ***n***  **(men/women)** | **Genotype distribution**  ***n* AA/GA/GG (%)** | **MAF**  **(95% CI)** | BMI adjustment | | No BMI adjustment | | **OR_add_**  **(95% CI)** |
|  |  |  |  | ***p*_add_** | ***p*_dom_** | ***p*_add_** | ***p*_dom_** |  |
| **Controls** | 4,914  (2,286/2,628) | 4,415/488/11  (89.9/9.9/0.2) | 5.2  (4.8-5.6) |  |  |  |  |  |
| **T2D patients** | 3,558  (2,119/1,439) | 3,155/391/12  (88.7/11.0/0.3) | 5.8  (5.3-6.4) | 0.3 | 0.3 | 0.1 | 0.09 | 1.15  (0.97-1.36) |
| ***FDFT1***  **rs7001819** | ***n***  **(men/women)** | **Genotype distribution**  ***n* TT/TC/CC (%)** | **MAF**  **(95% CI)** | BMI adjustment | | No BMI adjustment | | **OR_add_**  **(95% CI)** |
|  |  |  |  | ***p*_add_** | ***p*_dom_** | ***p*_add_** | ***p*_dom_** |  |
| **Controls** | 4,859  (2,259/2,600) | 1,969/2,291/599  (40.5/47.2/12.3) | 35.9  (34.9-36.9) |  |  |  |  |  |
| **T2D patients** | 3,472  (2,063/1,406) | 1,389/1,636/477  (40.0/47.1/12.9) | 36.4  (35.3-37.6) | 0.9 | 0.9 | 0.9 | 0.9 | 1.00  (0.92-1.08) |

Data are number of subjects, divided into genotype groups (% in each group), and frequencies of the minor allele (MAF) in percentages. General linear models were used to compare genotype distributions between type 2 diabetes (T2D) patients and glucose tolerant controls. Data was analysed applying and additive (*p*_add_) and dominant (*p*_dom_) model, and adjustment for sex and age and for sex, age and BMI were introduced, respectively. The odds ratios (OR) and 95% CI are given for the additive model without adjustment for BMI.
